# Male partner involvement in the prevention of mother to child transmission of HIV infection in Mwanza Region, Tanzania

**DOI:** 10.11604/pamj.2017.27.90.8901

**Published:** 2017-06-06

**Authors:** Munda Elias, Elia John Mmbaga, Ahmed Abade Mohamed, Rogath Saika Kishimba

**Affiliations:** 1 Muhimbili University of Health and Allied Sciences (MUHAS), School of Public Health and Social Sciences, Dar es Salaam, Tanzania; 2 Tanzania Field Epidemiology and Laboratory Training Programme Dar es Salaam; 3 Tanzania Ministry of Health and Social Welfare, 6 Samora Machel Avenue, P.O. Box 9083, 11478 Dar es salaam

**Keywords:** Male partner involvement, pregnant women, HIV/AIDS, PMTCT, Tanzania

## Abstract

**Introduction:**

Globally, there are 3.3 million children < 15 years of age living with HIV infection. About 95% of HIV infected children have acquired infection from their mothers. Although new pediatric HIV infection in Tanzania has declined by 48% and Prevention of Mother to Child Transmission (PMTCT) coverage of highly active anti-retroviral therapy (HAART) has increased to 77%, the MTCT rate remains high (15%). Poor male partner involvement in PMTCT services is one of the factors contributing to reduced effectiveness of the PMTCT and hence failure to achieve the elimination of maternal to child transmission of HIV. This study examined the predictors of male involvement in PMTCT services in Mwanza Region, Tanzania from perspectives of the mother.

**Methods:**

A cross sectional study involving selected health facilities was conducted in Mwanza urban from October 2013 through January 2014. HIV positive pregnant women attending ante-natal clinic (ANC) were interviewed using a semi structured questionnaire. Univariate analysis was used to describe the study respondents where bivariate and logistic regression was used to determine predictors of male involvement.

**Results:**

A total of 300 HIV positive mothers attending ANC with the mean age of 27.5 + 5.6 were interviewed. Few mothers (24.7%) had their male partners involved in PMTCT. Predictors of male partner involvement in PMTCT were mothers being proactive (Adjusted Odds Ratio (AOR) 28.6; Confidence Interval (CI) 7-116), perceived partners knowledge on PMTCT (AOR 24.6, CI 5.9-102.8), exposure to TV/Radio announcements on PMTCT (AOR 4.6, CI 1.5-14) and married status of the mother (AOR 3.7, CI 1.5-9). Mothers who never wanted to be escorted by their male partners and busy partners were associated with reduced odds of male involvement into PMTCT (AOR 0.07, CI 0.007-0.68) and (AOR 0.46 CI 0.21-0.99) respectively. Male partner involvement was associated with 98% reduced odds of violence (Crude Odds Ratio 0.018 CI 0.002-0.14).

**Conclusion:**

Male partner involvement in PMTCT is still low in Mwanza Region. Proactive mothers, partner's knowledge on PMTCT and announcements from television/radio were the major facilitating factors for male involvement in PMTCT as perceived by mothers. Busy male partners and mothers who did not want to be escorted by their partners were a hindrance to male involvement in PMTCT services. These factors highlight the importance of women role in promotion of PMTCT male involvement.

## Introduction

HIV and AIDS continue to be a major public health problem where almost 70 million people have been infected with the Human Immune deficiency Virus and about 35 million people have died of AIDS since the beginning of the epidemic [1]. Globally, there are 3.3 million children under fifteen years living with HIV infection whose 95% of them acquired the infection through Mother to Child Transmission (MTCT) [2]. Without intervention the rate of MTCT cumulatively from pregnancy to cessation of breastfeeding ranges from 25 to 48% [2–4]. Prevention of Mother To Child Transmission (PMTCT) program focuses on HIV pre and post test counseling, feeding option and use of Highly Active antiretroviral Therapy (HAART) during pregnancy, intrapartum and postpartum. Studies has shown that use of Anti Retro Viral drugs (ARV) can significantly reduce MTCT of HIV to less than 2% [5]. The 2012 WHO guideline recommends that countries with limited laboratory and infrastructure capacity to provide universal access to CD4 cell count testing, all confirmed HIV-infected pregnant and breastfeeding women must be offered life¬long triple ART as soon as diagnosed regardless of their CD4 count or clinical stage as part of the new Option B+ [6]. Success in preventing MTCT will not only depend on use of antiretroviral prophylaxis, but also on continuing support to the nursing mothers from their male partners. It has been shown that male partner involvement reduces the risk of vertical transmission and infant mortality by 40% compared to non-involvement [7]. Mechanism by which male involvement contributes to the effectiveness of PMTCT include male socio cultural superiority and influence in many of the Africa settings [8]. Women learn and retain more information when educated together with their partners [9] and some pregnant mothers don't accept HIV testing till they have their partners consent or assent [10]. Indeed utilization of PMTCT services by pregnant women is affected by several factors such as fear of disclosure of HIV results, lack of male partner support, fear of violence, abandonment and stigmatization which all involve a male partner. Male involvement facilitates both ART initiation and adherence [11, 12], it increases the probability of mothers delivering at health facility [13] and it enables for a good choice of breastfeeding plan [14–16]. This study aimed in determining magnitude, predictors as perceived by pregnant mothers and effects of male partner involvement in PMTCT services.

## Methods

This was hospital based cross sectional study involving HIV positive pregnant women in Mwanza urban conducted from October 2013 through January 2014. Mwanza urban has a population of 706,453 people where 237,478 are women in reproductive age group of 15 to 49 years [17, 18]. PMTCT services in the region started in 2004 at Nyamagana hospital and currently there are 45 health facilities which provide PMTCT services in the region. Ten out of 45 Health facilities in Mwanza urban were selected by simple random sampling using lottery method. Proportional to size sampling technique was used to obtain the number of study participants from each participating health facility (The size of each facility was the number of PMTCT attendees as per quarter of July-September 2013). We calculated the sample size based on the estimation of male involvement (defined as men who tested for HIV with their spouse at ANC) in the population of 21% [19], an absolute precision of 6%, design effect of 1.5 [17] and a 5% level of significance. We adjusted the sample size by considering 10% of anticipated non response. Therefore the total sample size was estimated at 300 HIV positive pregnant women. All pregnant mothers with at least 18 years who tested for HIV and agreed to be enrolled into PMTCT program were included. A pregnant mother in first or second trimester or if her partner is not alive was excluded from the study. Eligible clients from each Health facility who attended on days of interview were enrolled sequentially into the study. A face to face interview was conducted using a pre tested, semi structured questionnaire to collect information on socio demographic information both for participants and their male partners, partner testing and providing support to the mother and Factors affecting male participation in various PMTCT services as perceived by the mothers. Information on mother disclosure of HIV test result to her partner and any negative consequences after disclosure was also obtained.

Data were entered, cleaned, analyzed using Epi info version 3.5.4. Male partner involvement was a composite measure that included partner testing for HIV, partner support and partner aware of Mother's PMTCT status. Partner aware of mother's HIV status (disclosure) was further evaluated for existence of any form of Gender Based Violence. All independent variables including partner's knowledge were factors perceived by the mother as the respondent. Marital status such cohabiting and married were codes as living together while single and divorce were coded as not living together. Further coding for marital status was married for married couple while cohabiting, divorce and single were coded as not married. Univariate and Bivariate were done. Odds Ratio (OR) was used to quantify the likelihood of Male partner involvement into PMTCT and statistical significance was assessed using P- value and 95% confidence intervals for the odds ratios (OR). All independent variables with P-value <0.2 in the Bivariate analysis were considered for multivariate analysis through logistic regression to assess the effect of each covariate in the final model. Step down procedure for logistic regression was used to identify variables associated with male partner involvement in the final model. Ethical clearance was obtained from the Research Ethics Committee of Muhimbili University of Health and Allied Sciences. At the local level permission was also sought from Mwanza region authority and the respective health facilities authority. A written consent was obtained from each participant before interview.

## Results

### Socio demographic information

A total of 300 HIV positive pregnant mothers were interviewed in this study. The mean age of the respondents was 27.5 years with standard deviation of 5.57 years. Majority, 211 (70.3%) of these mothers were married. Median gravidity was three with Inter-quartile range (IQR) of two and four while the median number of living children was two with IQR of one and three. The illiterate rate among study participants was more than 10% (Table 1).

**Table 1:** Socio demographic characteristics of the study respondents (N=300)

Variable	Description	Respondents	
Age group (yrs)		**N**	**(%)**
	<26	110	36.6
	26-35	164	54.7
	36-45	26	8.7
	>45	0	0
Highest level of education attained			
	None	31	10.3
	Primary	173	57.7
	Secondary	77	25.7
	Tertiary	19	6.3
Marital status			
	Single	65	21.7
	Married	211	70.3
	Cohabiting	18	6
	Divorced	6	2
Gravidity			
	Prime	43	14.3
	Gravida two	49	16.3
	Gravida three	96	32
	Gravida four	62	20.7
	Grand multipara	50	16.7
Occupation			
	House wife	83	27.7
	Peasant	23	7.7
	Small scale business	110	36.7
	Self employment	31	10.3
	Employed	47	15.7
	Others	6	2
Religion			
	Christian	183	61
	Moslem	102	34
	Others	15	5
ANC visits			
	Twice	99	33
	Thrice	155	51.7
	Four times	44	14.7
	Five times	2	0.7

### Level of male partner involvement into PMTCT services

The level of male partner involvement in PMTCT was assesed using a series of questions as indicated in Table 2. Out of 300 participants, only 112 (37%) mothers had shared their HIV status with their partners. Partner`s HIV testing level was 114 (38%) of mothers reported their partners to have tested. A total of 96 (32%) mothers reported to have ever received support from their partners during the index pregnancy. The overall male partner involvement in PMTCT services (include men who scored yes in all the three questions) was 24.7% (74).

**Table 2:** Level of male partner involvement into PMTCT services (N=300)

Variables	Respondents’ response	
	n (%)	
	YES	NO
Male partner aware of PMTCT status of the mother	112 (37.3)	188 (62.7)
Male partner tested for HIV	114 (38)	186 (62)
Male partner provides support to the mother	96 (32)	204 (68)
Overall Male involvement	74 (24.7)	226 (75.3)

### Magnitude of gender based violence

**Figure 1:**
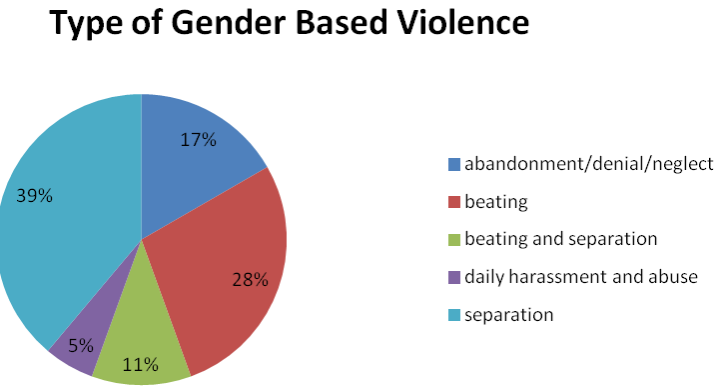
Type and magnitude of gender violences

Of the 112(37%) mothers who reported to have shared their HIV serostatus with their male partners 18 (16.1%) reported different types of gender abuse. A substantial proportion (39%) of women who experienced gender based violence reported that their partners decided to stay away from them (separation) and 11% stated that they were beaten before their partners went to live away. Beating alone was the second most common form of abuse (Figure 1).

### Predictors of male involvement in PMTCT services

Bivariate analyses were done in order to determine whether perceived factors by mother such as TV/Radio announcement, distance from home to ANC, ANC friendly services, socio cultural norms, mother being proactive, partner knowledge on PMTCT, willingness of a mother to be escorted to ANC and busy partner are associated with male partner involvement in Prevention of Mother to child transmission of HIV infection. Socio-demographic factors of both mother and partner such as marital status, partner being in same religion, polygamist partner and partner's level of education were also assessed. In the bivariate analysis, mothers who reported that their partners have been exposed to TV/Radio announcement addressing PMTCT had three times higher odds of their partners being involved in PMTCT than those who reported no such exposure (COR 2.9 CI 1.4-6.6). Likewise mothers who reported that their partner had knowledge on PMTCT had 20 times higher odds of their partners being involved in PMTCT than those who reported no such knowledge (COR 20.5 CI 5.8-72.8). Mothers who were asking for escort to ANC from their partners were having 38 times more chances for their partners to be involvement in PMTCT services than those were not asking for escort (COR 37.9 CI 11.0-130.6). In additional mothers who live together with their male partners are three times more likely to have their male partners involved in PMTCT services than those who don't live together (COR 3.2 CI 1.5-7.0) while mothers who are married have two fold more likely to have their partners involved in PMTCT services than those who are not married (COR 2.3 CI 1.2-4.6). Also mothers who reported to be in same religion with their partners had six times increased chances of male partner being involved in PMTCT services than those who were not in the same religion (COR 5.7 CI 1.7-19.0). On contrary, mothers who did not want to be escorted to ANC by their partners had 92% less likely to have their male partners involved in PMTCT services (COR 0.08 CI 0.01-0.6). Additionally mothers who reported their male partners to be busy with other daily activities especially on days of ANC had 62% less likely their male partners to be involved in PMTCT services (COR 0.38 CI 0.2-0.7). Furthermore socio-cultural norms and multiple marriages were limiting factors for male involvement in PMTCT [(COR 0.3 CI 0.1-0.9) and (COR 0.44 CI 0.2-0.9)]. Partners who had secondary education and above were 32% less likely to be involved in PMTCT services than those who had primary education and below however this association was not significant (Table 3). On logistic regression, proactive mothers who always ask for escort from their partners ranked the first as a promoting factor for male involvement in PMTCT services. Data show that mothers who always ask for escort from their partners had 28.6 times increased odds of their male partners being involved in PMTCT (AOR 28.6 CI 7.1-116). The second promoting factor for male involvement in PMTCT was perceived partner's knowledge on PMTCT. Data show that mothers who reported that their male partners being knowledgeable on PMTCT had 24.6 increased odds of their male partners being involvement in PMTCT than those mothers whose partners had no such knowledge (AOR 24.6 CI 5.9-102). Mothers whose partners had exposure to TV/Radio announcement addressing PMTCT had 4.6 times increased odds of their male partners being involved in PMTCT than those with no such exposure (AOR 4.6 CI 1.5-14.2) while married status of mother had 3.7 times more likely to have their male partners involved in PMTC (AOR 3.7 CI 1.5-9.4). On the other hand, Mothers who did not want to be escorted by their male partners were 93% less likely to have their male partners involved in PMTCT services than those who wanted escort (AOR 0.07 CI 0.01-0.7) while perceived busy partners were 54% less likely to be involved in PMTCT (AOR 0.46 CI 0.2-0.9). The rest factors which were significant on Bivariate analysis were not significant on logistic regression model (Table 3).

**Table 3:** Determinants of male partner involvement into PMTCT

	Male involvement		Crude Odds Ratio	Adjusted Odds Ratio
	YES	NO	(95% CI)	(95% CI)
TV/RADIO Enouncement				
Yes	13	15	2.99	4.6
No	61	211	(1.35-6.64)	**(1.5-14.17)**
Mother doesn’t want escort				
Yes	1	32	0.08	0.07
No	73	194	(0.01-0.62)	**(0.007-0.68)**
Polygamist partner				
Yes	8	49	0.44	0.35
No	66	177	(0.19-0.97)	(0.1-1.2)
Busy partner				
Yes	29	142	0.38	0.46
No	45	84	(0.22-0.65)	**(0.21-0.99)**
Partner having knowledge on PMTC				
Yes	16	3	20.5	24.6
No	58	223	(5.78-72.76)	**(5.9-102.8)**
Socio cultural norms				
Yes	5	41	0.3	0.43
No	69	185	(0.12-0.86)	(0.14-1.3)
Live together				
Yes	66	163	3.19	2.9
No	8	63	(1.45-7.02)	(0.9-8.8)
Same religion				
Yes	71	182	5.7	6.3
No	3	44	(1.72-19.02)	(0.9-40)
I always ask him to escort me				
Yes	25	49	37.9	28.6
No	3	223	(11.0-130.6)	**(7.05-116.1)**
Partners Education				
Secondary and above	34	125	0.68	1.1
Primary and below	40	101	(0.4-1.16)	(0.4-2.8)
Married				
Yes	61	150	2.3	3.7
No	13	76	(1.2-4.6)	**(1.49-9.38)**
ANC is too far				
Yes	2	1	6.2	1.7
No	72	225	(0.56-69.9)	(0.06-42.8)

### Effect of male partner involvement on domestic partner violence

In this study we aimed also at determining the effect of male partner involvement on reported gender based violence. There is a great association of reported gender based violence and male partner involvement as data show that those mothers whose partners are involved into PMTCT are 98% less likely to be involved in violence than those whose partners are not involved into PMTCT (Crude OR 0.02 P < 0.001) (Table 4).

**Table 4:** Association of male involvement and gender based violence (n=112)

	Gender based violence		OR	(95% CI)	P value
Male involvement	**YES**	**NO**			
YES	1	17	0.018	(0.002-0.14)	<0.001
NO	72	22			

## Discussion

This study was able to determine magnitude of male partner involvement into PMTCT services as well as identifying factors affecting male partner involvement into PMTCT services as perceived by mothers. Furthermore it has demonstrated that male partner involvement has a significant influence on preventing Gender based violence. The magnitude of male partner involvement shown in this study was 24.7%. This findings correlates with that reported by Byamugisha et all in Uganda where 26% of men whose wives were attending ANC at Mbale Regional Referral hospital reported to have full male involvement [20]. Likewise, the magnitude of male involvement in this study is more or less the same as that reported in the bottleneck analysis report of Tanzania e-MTCT of 2012 which showed only 21% male partner involvement [21], this slight difference might be attributed to under reporting of the routine data used to compile the bottleneck report. However due to differences in definition of male involvement and setting of the study, the magnitude in this study is much higher from 12.5% which was reported by Msuya SE et al in Moshi urban [22]. Studies done in Cameroon, South Africa and Malawi have reported male involvement of less than 20% [23–25]. In the same way as found by Kilewo C. et al in 2001 among women participating in a perinatal trial in Dar es salaam, less than 20% of them had shared their HIV results with their partners eighteen months after diagnosis [26]. This correlates with the findings of this study where the proportion of HIV positive pregnant mothers who have shared their serostatus to their partners was 37%. This could be contributed by low (38%) HIV testing level of male partners as noted also by Falnes EF et al 2010 in northern Tanzania [27]. Tadesse E et al 2004 in Blantyre Malawi found also that women have great trust to their male partners if and only if they are tested for HIV because majority of them choose their partner as their primary confidants after they tested for HIV [28].

In this study, it has been shown that women have a great role to play in making their male partners involved into PMTCT. Data show that proactive mothers who always ask their partners for escort to ANC are mostly associated with positive impact on male partner involvement. The studies done by Fisaha H et al 2012 in Makelle, Nothern Ethiopia on male partner involvement in PMTCT also found that maternal willingness to inform their husband about availability of testing services at ANC was an independent predictor for male partner involvement [29]. As expected, male partners who are knowledgeable on PMTCT are more likely to adopt PMTCT services. Data in this study reveals that male partners who are knowledgeable on PMTCT are 24 fold more likely to be involved into PMTCT than those who have no knowledge about PMTCT. Many studies have found that lacking PMTCT knowledge among male partners is a major hindrance to their male involvement [29, 30]. Furthermore, this study shows that TV/Radio broadcasts play a major role in encouraging men for PMTCT involvement. This is likely the main source of educating and promoting knowledge of PMTCT in the society. On the other hand factors such as socio cultural norms, polygamist partner, and perceived ANC distance which have been shown by other studies as determinants of male involvement in PMTCT [23, 31, 32] were not found to be significant in this study. This is likely to be due to changes in taboos favored by technological advancement in information sharing. Many people in urban area own TV, Radio and phones which have access to various communication media such as facebook, whats up, twitter to mention a few. People can acquire knew knowledge easily and fast, and this facilitates changes in taboos. Factors like friendly ANC services and partner fearing of HIV test result were inconclusively significant because of having empty numbers in some of the shells. This could require more exploration with large sample size. Male partner involvement into PMTCT services in its real definition as defined by a researcher has a negative impact on Gender Based Violence. In this study, male involvement was associated with reduced odds of GBV by 98%. Likewise, a study done by Semrau K et al in 2005 reported that couple counselling makes a man more responsible for the health of the partner and the family resulting to less blame and discrimination [33]. This indicates that counselling and HIV status disclosure resulting from male involvement reduce the probability for a male to engage in violence.

Despite that to a large extent this study has been able to answer intended research questions also it has some limitations. First in this study male information was extracted using female partner. A use of this approach in one way or another might affect the result in this study because female partner is more likely to have recall bias however the findings of this study correlate with other studies which interviewed men [20, 30]. Second the possibility of desirability bias is also expected because mothers attending PMTCT services are trained on PMTCT hence they tend to answer what they think is expected from them. Thirdly, the cross section nature of this study will not prove if the associated factors of male involvement are actually causal. However, most factors identified are also supported by robust studies done elsewhere. Fourth, we anticipated that the use of pre-prepare structure questionnaire may results into information bias, to minimizes this all interviewers were oriented to the meaning and intention of every question before beginning the study. Finally this study was done into a single selected region in Tanzania this might limit generalizability of its findings.

## Conclusion

This study was set out to demonstrate the magnitude of male partner involvement and measure the strength of association with the determinants. Male partner involvement into PMTCT services in Mwanza urban is low. Proactive mothers on asking escort from their partners is the mostly associated promoting factor of male involvement followed by partners' knowledge on PMTCT then hearing TV/Radio announcements addressing PMTCT. Unwillingness of a mother to be escorted by partner is the major hindrance of male involvement into PMTCT services. Male partner involvement is likely to reduce events of gender based violence. TV/Radio announcements addressing PMTCT need to be advocated so as to increase exposure to every man. Likewise women have a role to play in making their men participate in PMTCT activities.

### What is known about this topic

- Without intervention the rate of MTCT cumulatively from pregnancy to cessation of breastfeeding ranges from 25 to 48% [**2–4**];
- Studies have shown that use of Anti Retro Viral drugs (ARV) during pregnancy can significantly reduce MTCT of HIV to less than 2% [**5**];
- Success in preventing MTCT will not only depend on use of antiretroviral prophylaxis, but also on continuing support to the nursing mothers from their male partners. It has been shown that male partner involvement reduces the risk of vertical transmission and infant mortality by 40% compared to non-involvement [**7**].

### What this study adds

- This study demonstrates the magnitude of male partner involvement and its determinants in PMTCT. Proactive mothers on asking escort from their partners is the mostly associated promoting factor of male involvement followed by partners' knowledge on PMTCT then hearing TV/Radio announcements addressing PMTCT;
- Unwillingness of a mother to be escorted by partner is the major hindrance of male involvement into PMTCT services;
- Male partner involvement is likely to reduce events of gender based violence.

## Competing interests

The authors declare no competing interest.

## References

[1] UNAIDS (2012). Report on the global AIDS epidemics.

[2] Kourtis AP, Francis K, Abrams E (2006). Mother-to-child transmission of HIV-1: timing and implications for prevention. The Lancet.

[3] Teasdale C, Marais B, Abrams E (2011). Prevention of mother-to-child transmission. Journal of Clinical Evidence.

[4] De Cock KM, Fowler MG, Mercier E, De Vincenzi I, Saba J, Hoff E (2000). Prevention of mother-to-child HIV transmission in resource-poor countries: translating research into policy and practice. The Journal Of The American Medical Association.

[5] Lallemant M, Jourdain G, Le Coeur S (2004). Single-dose perinatal nevirapine plus standard zidovudine to prevent mother-to-child transmission of HIV-1 in Thailand. The new England Journal of Medicine.

[6] World Health Organisation (WHO) (2012). Use of ARV for treating pregnant women and preventing HIV infection in infants.

[7] Aluisio A, Richardson BA, Bosire R (2011). Male antenatal attendance and HIV testing are associated with decreased infant HIV infection and increased HIV-free survival. Journal of Acquired Immune Deficiency Syndromes.

[8] Sternberg P, Hubley J (2004). Evaluating men's involvement as a strategy in sexual and reproductive health promotion. Oxford journal; Health Promotion International.

[9] Mullany BC, Lakhey B, Shrestha D, Hindin MJ, Becker S (2009). Impact of husbands' participation in antenatal health education services on maternal health knowledge. JNMA; journal of the Nepal Medical Association.

[10] Bolu O, Allread V, Creek T, Stringer E, Forna F, Bulterys M (2007). Approaches for scaling up human immunodeficiency virus testing and counseling in prevention of mother-to-child human immunodeficiency virus transmission settings in resource-limited countries. American Journal of Obstetric & Gynaecology. Suppliment to September.

[11] Becker S, Mlay R, Schwandt HM, Lyamuya E (2010). Comparing couples' and individual voluntary counseling and testing for HIV at antenatal clinics in Tanzania: a randomized trial. Pubmed;Aids and Behavior joural.

[12] Conkling M, Shutes EL, Karita E, Chomba E, Tichacek A, Sinkala M (2010). Couples' voluntary counselling and testing and nevirapine use in antenatal clinics in two African capitals: a prospective cohort study. Journal of International AIDS Society.

[13] Kenya (2009). Doting dads can lower their children's HIV risk.

[14] Farquhar C, Kiarie JN, Richardson BA, Kabura MN, John FN, Nduati RW (2004). Antenatal Couple Counseling Increases Uptake of Interventions to Prevent HIV-1 Transmission. Journal of Acquired Immune Deficiency Syndrome.

[15] Bil SC, Otieno-Nyunya BA, Siika, Rotich JK (2008). Infant feeding practices among HIV infected women receiving prevention of mother-to-child transmission services at Kitale district hospital, Kenya. East African Medical Journal.

[16] Maru S, Datong P, Selleng D, Mang E, Inyang B, Ajene A (2009). Social determinants of mixed feeding behavior among HIV-infected mothers in Jos, Nigeria. Journal of AIDS Care.

[17] The United Republic of Tanzania (2012). Population and Housing census.

[18] Tanzania National Bureau of Statistics (2010). Tanzania Demographic health survey.

[19] Tanzania Minstry of Health and Social Welfare (2012). Elimination of mother to child transmission of HIV costed Plan from 2012-2015.

[20] Byamugisha R, Tumwine JK, Semiyaga N, Tylleskär T (2010). Determinants of male involvement in the prevention of mother-to-child transmission of HIV programme in Eastern Uganda: a cross-sectional survey. BioMed Central journal; Reproductive Health.

[21] Tanzania Ministry of Health and Social Welfare (2012). Bottleeneck analysis for virtual elimination of Mother To Child Transmission in Tanzania.

[22] Msuya SE, Mbizvo EM, Hussain A, Uriyo J, Sam NE (2008). Low male partner participation in antenatal HIV counselling and testing in northern Tanzania: implications for preventive programs. Journal of AIDS care.

[23] Nkuoh GN, Meyer DJ, Tih PM, Nkfusai J (2010). Barriers to men's participation in antenatal and prevention of mother-to-child HIV transmission care in Cameroon. Africa Journal of midwifery & women's health.

[24] Peltzer K, Mosala T, Dana P (2008). Follow-up Survey of women who have undergone a Prevention of Mother-to-Child Transmission Program in a resource-poor setting in South Africa. Journal of the Association of Nurses in AIDS Care.

[25] Kamanga E, Mofolo I, Martinson F, Pagadala N, Mwale G, Murotho J Challenges of male involvement in PMTCT.

[26] Kilewo C, Massawe A, Lyamuya E, Semali I, Kalokola F, Urassa E (2001). HIV counseling and testing of pregnant women in sub-Saharan Africa: experiences from a study on prevention of mother-to-child HIV-1 transmission in Dar es Salaam, Tanzania. Journal of Acquired Immune Deficiency Syndromes.

[27] Falnes EF, Tylleskär T, de Paoli M, Manongi R (2010). Mothers' knowledge and utilization of prevention of mother to child transmission services in Northern Tanzania. Journal of International AIDS Society.

[28] Tadesse E, Muula AS, Misiri H (2004). Likely stakeholders in the prevention of mother to child transmission of HIV/AIDS in Blantyre, Malawi. Africa Health Science.

[29] Fisaha H, Yemane B (2014). Male involvements in PMTCT: a cross sectional study, Makelle, Nothern Ethiopia. BMC Journal of pregnancy and childbirth.

[30] Theuring S, Mbezi P, Jordan B, Kunz A, Harms G (2009). Assessment on male partner involvement in ANC/PMTCT services in Mbeya region, Tanzania. Journal of AIDS Behaviour.

[31] Paek HJ, Lee B, Salmon C (2008). The contextual effects of gender norms, communication, and social capital on family planning behaviors in Uganda: a multilevel approach. Health Educ Behav.

[32] Boniface Y (2010). Willingness and participation towards PMTCT among males of reproductive age: study from Kilimanjaro. Dar Es Salaam Medical Students' Journal.

[33] Semrau K, Kuhn L, Vwalika C, Kasonde P, Sinkala M, Kankasa S (2005). Women in couples antenatal HIV counseling and testing are not more likely to report adverse social events. Journal of Acquire Immune deficiency Syndrome.

